# Inhibition of Dopamine Activity and Response of *Rhipicephalus microplus* Challenged with *Metarhizium anisopliae*

**DOI:** 10.3390/jof8121312

**Published:** 2022-12-17

**Authors:** Victória Silvestre Bório, Thaís Almeida Corrêa, Jéssica Fiorotti, Emily Mesquita, Laura Nóbrega Meirelles, Mariana Guedes Camargo, Vânia Rita Elias Pinheiro Bittencourt, Patrícia Silva Golo

**Affiliations:** 1 Programa de Pós-Graduação em Ciências Veterinárias, Instituto de Veterinária, Universidade Federal Rural do Rio de Janeiro, Seropédica 23897-000, Brazil; 2 Departamento de Bioquímica e Imunologia, Faculdade de Medicina de Ribeirão Preto, Universidade de São Paulo, São Paulo 14040-900, Brazil; 3 Departamento de Parasitologia Animal, Instituto de Veterinária, Universidade Federal Rural do Rio de Janeiro, Seropédica 23897-000, Brazil

**Keywords:** dopamine receptor antagonist, entomopathogenic fungi, biological control, hemocytes, phagocytic activity, ticks

## Abstract

Dopamine modulates ticks and insect hemocytes and links these arthropods’ nervous and immune systems. For the first time, the present study analyzed the effect of a dopamine receptor antagonist on the survival, biological parameters, phagocytic index, and dopamine detection in the hemocytes of ticks challenged by *Metarhizium anisopliae*. The survival and egg production index of *Rhipicephalus microplus* were negatively impacted when ticks were inoculated with the antagonist and fungus. Five days after the treatment, the survival of ticks treated only with fungus was 2.2 times higher than ticks treated with the antagonist (highest concentration) and fungus. A reduction in the phagocytic index of hemocytes of 68.4% was observed in the group inoculated with the highest concentration of the antagonist and fungus compared to ticks treated only with fungus. No changes were detected in the *R. microplus* levels of intrahemocytic dopamine or hemocytic quantification. Our results support the hypothesis that dopamine is crucial for tick immune defense, changing the phagocytic capacity of hemocytes and the susceptibility of ticks to entomopathogenic fungi.

## 1. Introduction

The hard tick *Rhipicephalus microplus* feeds preferably on bovines. Due to their hematophagous habit, they play a crucial role in the transmission of pathogenic agents such as the protozoa *Babesia* spp. and the bacterium *Anaplasma* [1]. The control of these ectoparasites is mainly performed using synthetic acaricides that, when inappropriately used, cause the selection of resistant populations, and can also contaminate the environment and animal products [2,3,4]. Biological control using entomopathogenic fungi is a promising alternative to control *R. microplus*, and the entomopathogenic fungus *Metarhizium* has proven to be highly effective against ticks [5,6]. Its spores adhere to the tick cuticle, penetrates, and multiplies inside, leading to the host’s death [7].

In the field, the biological control of ticks using fungi is challenged by the time required to kill these parasites and by the need for high concentrations of fungal propagules in comparison to insect trials. In addition to abiotic factors, the success of tick control using fungi depends on the interaction between this arthropod’s immune system and the fungal pathogen. Understanding the mechanisms of this interaction will help to enhance the use of entomopathogenic fungi as effective acaricides.

Arthropods have defenses against the infectious agents that affect them, including entomopathogenic fungi. The physicochemical and physiological barriers, such as the cuticle, the intestinal barrier, and cellular and humoral interactions, are examples of defense mechanisms [8,9,10]. Hemocytes are cells present in the hemolymph of ticks that are similar to vertebrates’ blood cells, and are involved in the processes of phagocytosis, nodulation, and encapsulation [11,12,13]. Phagocytosis is generally the process of consuming invaders to remove them from circulation and is mediated by specialized cells [12]. In ticks, studies reported the capacity of hemocytes to phagocyte a variety of microbes, including bacteria, fungi, yeasts, spirochetes, and foreign particles [11,13,14,15]. Phagocytosis is considered the most important innate immune response in invertebrates [16], including ticks; it is mainly accomplished by plasmatocytes and granulocytes [9,11,17,18].

Hemocytes can also produce dopamine (DA), a biogenic monoamine that links the immune and nervous systems of arthropods [19,20]. In insects, DA is related to early hemocyte signaling, stimulating phagocytosis and total hemocyte count [21]. In ticks, it is already known that DA acts on saliva production [22], and that exotic DA supports *R. microplus* in the challenge with an entomopathogenic fungus [20]. However, studies are needed to completely elucidate the influence of DA on the immune response of ticks, particularly the immune response mediated by hemocytes. The knowledge of immune responses in arthropods comes especially from research on *Drosophila*, *Aedes*, and *Anopheles* [13]. Tick immunity still has many knowledge gaps and has been little explored [23]; therefore, studies targeting cellular immune signaling pathways and their connections are critical for a better understanding of tick–parasite interactions and advances in tick control.

Invertebrates have three classes of DA receptors: (1) D1-like receptors, (2) D2-like receptors, and (3) DA intracellular receptors. The first two classes are similar to vertebrate receptors [19]. In hemocytes of *Aedes aegypti*, the addition of the dopamine D1 receptor antagonist (i.e., SCH23390) strongly inhibited DA receptors [24]. In 2011, Meyer et al. [25] managed to clone and characterize two D1 dopamine receptors in *Ixodes scapularis* hemocytes. These and other authors characterized DA receptor antagonists in *I. scapularis* cells and performed comparative pharmacological analyses, showing that the antagonist SCH23390 is a common antagonist of the two dopamine receptors in these ticks [25,26]. However, to date, there are no assays involving DA receptors in *R. microplus*, especially when these ticks are challenged with entomopathogenic fungi.

In the present study, the inoculation of a DA receptor antagonist into *R. microplus* aimed to analyze the impact of this inhibition on phagocytosis, biological parameters, tick survival, quantification of hemocytes, and DA detection in hemocytes when ticks were challenged or not with the entomopathogenic fungus *Metarhizium anisopliae*. The results obtained in the present study can be used to better understand the immune response of ticks, particularly when treated with entomopathogenic fungi, allowing advances in the biological control of ticks, and revealing tick immune responses to pathogens.

## 2. Materials and Methods

### 2.1. Rhipicephalus microplus Ticks

Fully engorged *R. microplus* females were collected from the floor of cattle pens holding artificially infested calves at the Wilhelm Otto Neitz Parasitological Research Station at the Federal Rural University of Rio de Janeiro (UFRRJ), Brazil (CEUA/Veterinary Institute, UFRRJ, Seropédica, Brazil—protocol No. 9714220419). After collection, ticks were washed in tap water and immersed in 0.05% sodium hypochlorite solution for three minutes, then dried and identified.

### 2.2. Metarhizium anisopliae and Fungal Suspension

The isolate *M. anisopliae* sensu stricto LCM S04 [27] was used to treat the ticks. This isolate was cultivated on an oat medium under controlled conditions (25 ± 1 °C; relative humidity (RH) ≥ 80) for 14 days and stored at 4 °C. The isolate was maintained in the Entomopathogenic Fungi Culture Collection of the Laboratory of Microbial Control (LCM S04, from Instituto Oswaldo Cruz, FIOCRUZ, under the code IOC 4694.). It was also deposited in the Filamentous Fungi Culture Collection (CCFF) at Instituto Oswaldo Cruz (FIOCRUZ) under the code IOC 4694. As the present study accessed the Brazilian genetic heritage, the research was registered in the National System for the Management of Genetic Heritage and Associated Traditional Knowledge (Sisgen) under code AA47CB6.

*M. anisopliae* conidia were added to a polyoxyethylene sorbitan monooleate (Tween 80, Vetec Fine Chemicals Ltd.a, Rio de Janeiro, RJ, Brazil) solution at 0.01% (*v/v*), vortexed for one minute for homogenization, quantified in a Neubauer chamber, and adjusted to 1.0 × 10^7^ conidia/mL. Prior to the bioassays, an aliquot of 10 μL of conidial suspension was transferred to potato dextrose agar (PDA) and incubated at 25 ± 1 °C and RH ≥ 80% to assess fungal viability. Conidia germination was determined 24 h after incubation.

### 2.3. Antagonist SCH 23390

SCH 23390 (Sigma-Aldrich, St. Louis, MO, USA) has been used in research with insects [19,26,28], and it has been shown to be an antagonist of dopamine receptors in ticks [26]. The antagonist was diluted in phosphate-buffered saline ((PBS) 0.13 M NaCl, 0.001 M KH_2_PO_4_, 0.02 M Na_2_HPO_4_, 0.003 M KCl, pH 7.2), and two concentrations were prepared, 1 nM and 1 µM, according to the manufacturer’s recommendations. The word “antagonist” in this text always refers to this antagonist of DA receptors.

### 2.4. In Vitro Phagocytic Assay

The phagocytic index (PI) was calculated through an in vitro assay using tick hemocytes collected from untreated females. Before the entire procedure, circular coverslips were placed in a 24-well plate (Kasvi, São José dos Pinhais, PR, Brazil). The hemolymph from 200 fully engorged tick females was collected [12] in 450 μL of L-15 Leibovitz Gibco (L-15) medium (Sigma-Aldrich, St. Louis, MO, USA) supplemented with 10% fetal bovine serum. Hemocytes were quantified in a Neubauer chamber, and approximately 2 × 10^4^ cells [20] were allocated to each well. After harvesting, hemocytes were submitted to different treatments.

Cells were exposed to the following treatments: (a) control (incubated without treatment) (CTR), (b) incubated with 10 µL PBS (PBS), (c) incubated with 10 µL of antagonist at 1 nM (SCH 1 nM), (d) 1 µM (SCH 1 µM), (e) 20 µL of *M. anisopliae* aqueous suspension at 1.0 × 10^7^ conidia/mL (MA), and (f) associations of antagonist and fungus (SCH 1 nM + MA and SCH 1 µM + MA). Zymosan A (*Saccharomyces cerevisiae)* (Sigma-Aldrich, St. Louis, MO, USA) (20 µL) at 1.0 × 10^7^ conidia/mL (Z) and associations of antagonist at 1 nM or 1 µM plus Zymosan (SCH 1 nM + Z or SCH 1 µM + Z) were used for phagocytic control.

First, cells received the antagonist and were incubated for one hour at 32 °C. After incubation, cells were exposed to *M. anisopliae* or Zymosan A, and the wells were completed with L-15 medium to a final volume of 250 µL. The 24-well plate was again incubated at 32 °C for two hours. The medium was then removed from the plate. Cells were fixed with 200 µL of methanol (Sigma-Aldrich, St. Louis, MO, USA) for three min and stained with 200 µL of Giemsa Sigma-Aldrich for 30 min [29]. Coverslips were washed with 200 µL of PBS and added to slides with the mounting medium. Hemocytes with internalized fungal propagules were counted at ×1000 magnification. The number of hemocytes with internalized propagules was obtained by counting 100 hemocytes on at least six slides. The entire experiment was performed three times with two independent replications.

### 2.5. Inoculation Treatments in Rhipicephalus microplus Females

Fully engorged *R. microplus* females were inoculated with the antagonist and the fungus to evaluate the ticks’ biological parameters and survival, quantify hemocytes, and detect DA in the hemocytes. Females were inoculated using a microinjector (Drummond, Broomall, PA, USA). The groups were the control without inoculation (CTR), inoculated with 276 nL of PBS (PBS), inoculated with 276 nL of the antagonist at 1 nM (SCH 1 nM), inoculated with 276 nL of the antagonist at 1 µM (SCH 1 µM), inoculated only with *M. anisopliae* suspension at 1.0 × 10^7^ conidia/mL (276 nL; ~2.760 conidia) (MA), inoculated with the antagonist at 1 nM followed by the fungal suspension (SCH 1 nM + MA), and inoculated with the antagonist at 1 µM followed by the fungal suspension (SCH 1 µM + MA). The antagonist solution was inoculated 20 min before the fungal suspension. After the treatments, the females were kept at 27 °C and RH ≥ 80% for 24 h.

### 2.6. Survival and Biological Parameters of Rhipicephalus microplus

The biological assay was performed with the groups described in item 2.5. The groups had ten females each with homogeneous weights. Tick females were kept at 27 °C and RH ≥ 80% throughout the experiment. Survival was analyzed daily for 10 days. In parallel, each female had their eggs weighed individually and daily. After death, the females were weighed individually.

The following biological parameters were analyzed: female’s initial weight (FIW), egg mass weight (EMW), female residual weight (FRW), and larval hatch (LH). The egg production (EPI) (EPI = EMW/FIW × 100) [30] and nutritional index (NI) (NI = EMW/FIW ‒ FRW × 100) [30] were also calculated. The reproductive efficiency (RE) (RE = EMW/FIW × LH × 20,000) was used to obtain the tick control percent [31], which was calculated in relation to the control group. The entire experiment was performed three times.

### 2.7. Quantification of Hemocytes

Tick females were treated according to item 2.5. Each group contained 30 females. Then, 24 h after the treatments, the hemolymph was collected [12] in the L-15 medium into iced microtubes. Each microtube with 100 µL of the medium received hemolymph collected from ten females. Quantification of the hemocytes was performed in a Neubauer chamber, and the volume of the medium was discounted. The experiment was carried out in triplicate, and the entire experiment was performed twice.

### 2.8. Dopamine Detection in Hemocytes of Rhipicephalus microplus

As the different concentrations of the antagonist did not yield different results in the phagocytic index, quantification of hemocytes, and ticks’ survival assays, for the detection of DA, only the highest concentration of the antagonist (1 µM) was used. Accordingly, four experimental groups were organized with 25 ticks each: untreated ticks (control group (CTR)), ticks inoculated with the antagonist at the highest concentration (SCH 1 µM), ticks inoculated with fungus (MA), and ticks inoculated with the antagonist and then the fungus (SCH 1 µM + MA). Females were inoculated according to item 2.5, and the experiment was performed 24 h after inoculation. Hemolymph was collected [12], placed in 500 μL of the L-15 medium, and the hemocytes were quantified in a Neubauer chamber. Circular coverslips were placed in a 24-well plate (Kasvi, São José dos Pinhais, PR, Brazil), and approximately 2 × 10^4^ cells were allocated to each well. Hemocytes were fixed with 4% paraformaldehyde for 30 min and washed in PBS three times. Hemocytes were incubated with anti-dopamine antibodies (ab6427; Abcam, Cambridge, UK) for 72 h, and with the secondary antibody SA-Alexa Fluor 594 for one hour. Nuclei of hemocytes were stained with DAPI (blue) at room temperature, and hemocytes were observed under a BX 51 fluorescence microscope (Olympus) according to the adapted protocol described by Wu et al. (2015) [19]. Fluorescence quantification was performed using the ImageJ 1.52 software (National Institute of Health, Bethesda, MD, USA) to calculate the area fraction intensity (%). The experiment was carried out in triplicate.

### 2.9. Statistical Analysis

All data were analyzed using GraphPad Prism version 8.0 for Windows (GraphPad Software, San Diego, CA, USA). The data were checked for normality using a Shapiro–Wilk test. The quantification of hemocytes, DA detection in the hemocytes, and phagocytic index data had normal distribution and were analyzed by a one-way ANOVA followed by Tukey’s test (*p* < 0.05). Tick survival was analyzed using the Log-rank test. Tick biological parameters data had non-normal distribution and were submitted to the Kruskal–Wallis test followed by the Dunn test (*p* < 0.05).

## 3. Results

### 3.1. Phagocytic Index of Rhipicephalus microplus Hemocytes Challenged with Metarhizium anisopliae and the Antagonist

**Figure 1:**
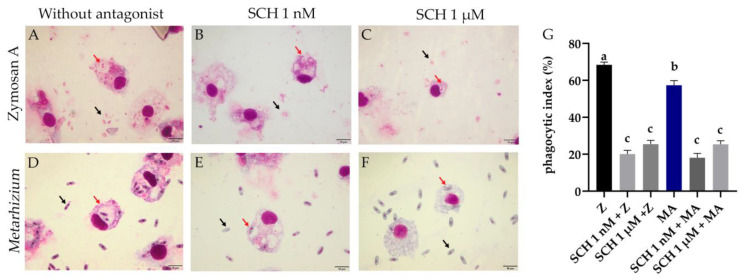
(**A**–**F**) *Rhipicephalus microplus* hemocytes on slides exposed to *Metarhizium anisopliae* (MA) or Zymosan (Z) for two hours with or without previous incubation of SCH 23390 dopamine receptor antagonist at 1 nM or 1µM for one hour. (**A**) Z; (**B**) SCH 1 nM + Z; (**C**) SCH 1 µM + MA; (**D**) MA; (**E**) SCH 1 nM + MA; (**F**) SCH 1 µM + MA. Black arrows indicate *Metarhizium* conidia or Zymosan that were not phagocytosed. Red arrows indicate conidia or Zymosan phagocytosed. The scale bar represents 10 μm. (**G**) Phagocytic index (%) of *R. microplus* hemocytes after incubation with *M. anisopliae* conidia or Saccharomyces cerevisiae (Zymosan A) with or without SCH 23390. Data were analyzed by one-way ANOVA and the Tukey’s test (*p* < 0.05). Different letters differ statistically. Z: cells exposed to Zymosan A alone; SCH 1 nM + Z: cells exposed to the antagonist at 1 nM followed by Zymosan A; SCH 1 µM + Z: cells exposed to the antagonist at 1 µM followed by Zymosan A; MA: cells exposed to *M. anisopliae* alone; SCH 1 nM + MA: cells exposed to the antagonist at 1 nM followed by *M. anisopliae*; SCH 1 µM + MA: cells exposed to the antagonist at 1 µM followed by *M. anisopliae*.

Phagocytic cells were counted on slides (Figure 1A), and 100 cells were counted per slide. The SCH 1 nM + Z, SCH 1 µM + Z, SCH 1 nM + MA, and SCH 1 µM + MA groups did not differ from each other. The Z (68.4%) and MA (57.3%) groups had the highest phagocytic indexes and were different from each other (*p* = 0.006). The associated groups SCH 1 nM + Z (20.1%), SCH 1 µM + Z (25.4%), SCH 1 nM + MA (18.1%), and SCH 1 µM + MA (25.3%) were statistically different from Z (*p* < 0.0001) and MA (*p* < 0.0001) (Figure 1G). The phagocytic index of hemocytes in the presence of the antagonist (independent of the addition of *Metarhizium* or Zymosan) was lower in all groups (*p* < 0.0001) when compared to the control groups (Z and MA) (Figure 1G).

### 3.2. Survival and Biological Parameters of Rhipicephalus microplus Females

**Figure 2:**
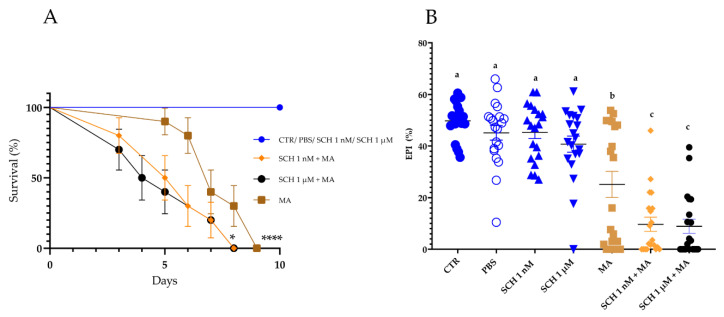
(**A**) Effect of dopamine receptor antagonist SCH23390 on the survival of *Rhipicephalus microplus* females associated or not with *Metarhizium anisopliae*. Mean survival (%) and standard deviation of females inoculated with *M. anisopliae* conidia according to Log-rank (*p* < 0.0001). A representative experiment of three independent replications, where (*) represents statistical difference between survival averages from MA and SCH 1 nM + MA (*p* = 0.0253) or MA and SCH 1 µM + MA (*p* = 0.0291), and (****) represents statistical difference between survival averages from MA and CTR (*p* < 0.0001) or MA and SCH 1 nM or SCH 1 µM (*p* < 0.0001). (**B**) Egg production index (EPI) of *R. microplus* females inoculated with antagonist SCH 23390 at 1 nM or 1 µM, *M. anisopliae* LCM S04 at 1.0 × 10^7^ conidia/mL and associations. Different letters differ statistically. CTR: untreated ticks; PBS: ticks inoculated with phosphate buffer solution; SCH 1 nM: ticks inoculated with antagonist at 1 nM; SCH 1 µM: ticks inoculated with antagonist at 1 µM; MA: ticks inoculated with *M. anisopliae*; SCH 1 nM + MA: ticks inoculated with the lowest concentration of antagonist and fungus; SCH 1 µM + MA: ticks inoculated with the highest concentration of antagonist and fungus.

Ticks in the control groups (CTR and PBS) and ticks inoculated with the antagonist alone (SCH 1 nM or SCH 1 µM) exhibited 100% survival for 15 days. The mean survival time of ticks inoculated with the antagonist and then the fungus (SCH 1 nM + MA (5.5 days) and SCH 1 µM + MA (4.5 days)) was lower (*p* = 0.025 and *p* = 0.029, respectively) than the survival of the group treated with *M. anisopliae* alone (MA (7 days)) (Figure 2A). CTR exhibited higher survival than MA (*p* < 0.0001) (Figure 2A).

The biological parameters that were analyzed are exhibited in Table S1. There was a significant reduction in the EPI of tick females inoculated with the antagonist followed by the fungus (SCH 1 nM + MA or SCH 1 µM + MA) in comparison to the other groups, including the group inoculated with *M. anisopliae* alone (Figure 2B; Table S1). The average EPI of CTR was 49.8 ± 1.5%, while in MA it was 25.1 ± 5.0%. SCH 1 nM + MA exhibited an average EPI of 9.6 ± 2.7% and SCH 1 µM + MA 8.9 ± 2.7% (Figure 2B). The tick control percent was higher in the fungus-treated groups (MA (78.2%), SCH 1 nM + MA (79.2%), and SCH 1 µM + MA (90.5%)) (Table S1).

CTR, PBS, SCH 1 nM, and SCH 1 µM yielded similar average egg mass weights, EPIs, NIs, and REs. These averages were higher than the ones yielded by the ticks treated with the fungus (previously inoculated or not with the antagonist) (Table S1). Accordingly, the administration of the antagonist (at the higher or lower concentration) did not change the biological parameters of the *R. microplus*’s analyzed here. Except for the EPI, the average EMWs, NIs, and REs from MA were similar to those observed in SCH 1 nM + MA and SCH 1 µM + MA (Table S1).

### 3.3. Quantification of Hemocytes

**Figure 3:**
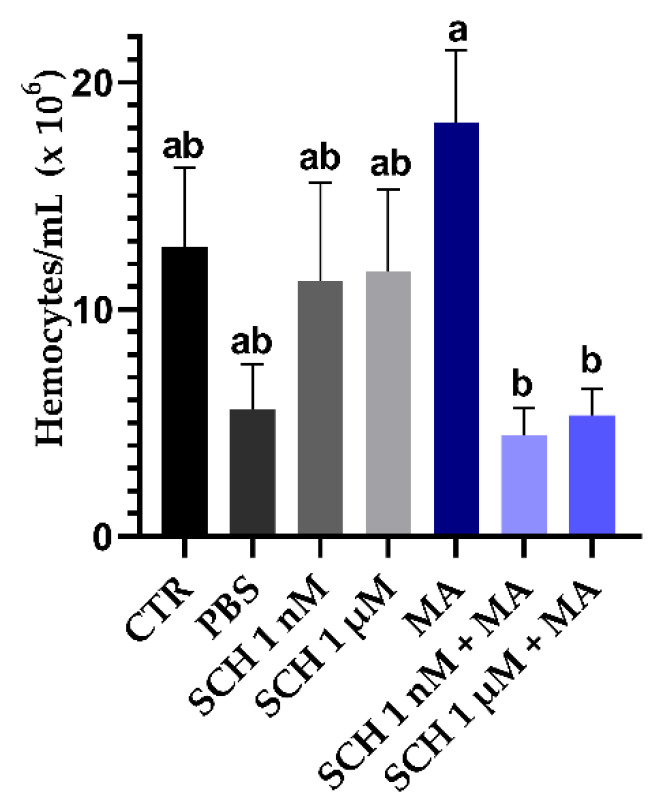
Average and standard error of *Rhipicephalus microplus* hemocytes circulating in the hemolymph 24 h after inoculation of the antagonist SCH 23390 and *Metarhizium anisopliae* LCM S04. Bars with the same letter do not differ statistically according to one-way ANOVA followed by Tukey’s test (*p* < 0.05). CTR: untreated ticks; PBS: ticks inoculated with phosphate buffer solution; SCH 1 nM: ticks inoculated with the antagonist at 1 nM; SCH 1 µM: ticks inoculated with the antagonist at 1 µM; MA: ticks inoculated with *M. anisopliae*; SCH 1 nM + MA: ticks inoculated with the lowest concentration of antagonist and fungus; SCH 1 µM + MA: ticks inoculated with the highest concentration of the antagonist and fungus.

Inoculation of the *M. anisopliae* LCM S04 conidia alone did not reduce circulating hemocytes in *R. microplus* (Figure 3). Females inoculated exclusively with the fungus (MA) had a higher number of circulating hemocytes (1.8 × 10^7^ hemocytes/mL) than females inoculated previously with the antagonist and then the fungus (SCH 1 nM + MA: 4.4 × 10^6^ hemocytes/mL; *p* = 0.029) (SCH 1 µM + MA: 5.3 × 10^6^ hemocytes/mL; *p* = 0.048). The inoculation of the antagonist at 1 nM or 1 µM did not change the number of circulating hemocytes (SCH 1 µM: 11.2 × 10^6^ hemocytes/mL; SCH 1 µM: 11.6 × 10^6^ hemocytes/mL) in comparison to untreated ticks (CTR: 12.7 × 10^6^ hemocytes/mL) (*p* = 0.999 and *p* > 0.999).

### 3.4. Dopamine Detection in R. microplus Hemocytes

**Figure 4:**
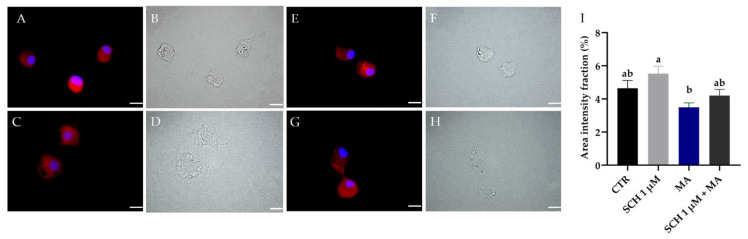
Detection of dopamine in the hemocytes of *Rhipicephalus microplus* tick females 24 h after inoculation of dopamine receptor antagonist. (**A**,**C**,**E**,**G**) Immunofluorescence images and (**B**,**D**,**F**,**H**) light microscopy images. (**A**,**B**) untreated ticks (CTR); (**C**,**D**) ticks inoculated with antagonist (SCH 1 µM); (**E**,**F**) ticks inoculated with *Metarhizium anisopliae* (MA); (**G**,**H**) ticks inoculated with antagonist followed by *M. anisopliae* (SCH 1 µM + MA). The scale bar represents 10 μm. (**I**) Average fluorescence intensity (marked area) percentage and standard error of dopamine in the hemocytes of *R. microplus* tick females. Bars with the same letter did not differ statistically according to one-way ANOVA and the Tukey’s test (*p* < 0.05).

As expected, DA could be detected in the hemocytes from all groups (including untreated ticks). The images in Figure 4 show DA granules labeled with the anti-DA antibody (red) in the cytosol of hemocytes, allowing the analysis of the presence of dopamine in the cells (Figure 4A–H). SCH 1 µM exhibited higher DA intensity (5.5% ± 0.4) than MA (3.4% ± 0.2) (Figure 4I). The area intensity fraction of DA from CTR (4.6% ± 0.4%) and SCH 1 µM + MA (4.2% ± 0.3%) was similar (*p* = 0.844).

## 4. Discussion

The biological control of ticks using entomopathogenic fungi is an alternative to the use of synthetic acaricides, and studies over the decades reported that this is a highly effective method [5,6,32,33]. Dopamine is a biogenic monoamine that links two of the most important systems of arthropods: immune and nervous [19,20]. Blocking the action of dopamine makes it possible to analyze the influence of the dopaminergic pathway on ticks’ susceptibility to pathogens, including fungi.

Recent studies reporting the mortality of *Ixodes ricinus* female ticks after treatment with entomopathogenic fungi showed survival averages of around 5 and 11 days with suspensions at concentrations of 2 × 10^6^ and 2 × 10^7^ conidia/mL, respectively [9]. A recent study evaluated the effect of exogenous DA on the action of entomopathogenic fungi [20]. These authors reported that ticks injected with DA and treated with *M. anisopliae* had higher survival than ticks inoculated exclusively with *M. anisopliae* [20]. As expected, in the present study, untreated females remained alive at least for 15 days, while the survival of ticks inoculated only with the fungus was 7 days on average. Following our initial hypothesis, tick females, previously inoculated with the dopamine receptor antagonist and then the fungus, died before the ones that did not receive the antagonist (Figure 2A). The inoculation of the antagonist alone did not change the biological parameters or the survival of ticks, suggesting that the variations observed here were due to the fungal infection. Our results also corroborate the assumption that DA has a key role in the immune response of ticks, since blocking a dopamine receptor reduced the survival of the females challenged with fungus. Although exogenous DA has been proven to increase *R. microplus* survival after the challenge with *M. anisopliae* [20], this is the first time that a study was conducted with a dopamine receptor antagonist and ticks to analyze the susceptibility of females to an entomopathogenic fungus.

In the present study, besides tick survival, the biological parameters of ticks were also analyzed. As expected, fungus-treated females (with or without the antagonist) exhibited lower biological parameters (Table S1). A comparison between MA and SCH 1 nM + MA or SCH 1 µM + MA showed that only the EPI was affected, not the EMW, NI, or RE (Table S1). Although both the EPI and NI consider the capacity of the tick female to produce eggs, the former does not include the final weight of the tick females after oviposition [30]. Accordingly, one can infer that ticks previously inoculated with the antagonist and then treated with fungus produced fewer eggs (i.e., lower EPI (Figure 2B)), and it probably happened because the females died faster and were not able to convert the blood in their intestine into eggs (i.e., lower tick survival (Figure 2A)). As the NI considers the final weight of the tick, and females that die faster tend to remain with their intestines partially full, no difference in the NI between MA and SCH + MA is expected.

Here, the quantification of hemocytes and their phagocytic index were analyzed to understand the effect of DA inhibition in the hemocytes of *R. microplus*. Contrary to what was expected (according to De Paulo et al. [34]), in the present study, the inoculation of fungus alone did not change the number of circulating hemocytes (CTR compared to MA) 24 h after treatment (Figure 4). De Paulo et al. [34] reported a reduction in the number of circulating hemocytes in *R. microplus* after fungal treatment. Nevertheless, these authors used different *Metarhizium* isolates and inoculation volume. Accordingly, our results suggested that different fungal species, isolates, and doses may trigger varied responses in ticks. In other words, as the cellular response is one of the responses to pathogens in ticks, the similar number of hemocytes observed in ticks from CTR and MA suggested that the lower number of propagules inoculated (in comparison to De Paulo et al. [9]) allowed a cellular response by the tick within 24 h. On the other hand, in the present study, the number of hemocytes reduced statistically when ticks inoculated only with fungus (MA) were compared to ticks inoculated with the antagonist and then the fungus (SCH 1 nM + MA or SCH 1 µM + MA) (Figure 4). The lower number of hemocytes in SCH 1 nM + MA and SCH 1 µM + MA compared to MA may be a consequence of the combined factors (DA inhibitor plus fungal action).

Corrêa et al. [20] reported the detection of DA in the hemocytes of *R. microplus* ticks under physiological conditions for the first time, suggesting these cells can naturally produce DA. The same results were reported in insects, and DA in the presence of the antagonist SCH 23390 was not detected [19]. The detection of dopamine in *R. microplus* hemocytes in the present study (Figure 4) confirmed the results of Corrêa et al. [20] and demonstrated that the administration of the antagonist SCH 23390 was not able to decrease or increase the DA detection in the hemocytes. However, hemocytes from ticks inoculated only with the antagonist had a higher average fluorescence intensity of DA than hemocytes from the MA group (Figure 4). The lower DA detection in MA compared to SCH could be related to the use (cellular release) of this monoamine for phagocytosis signaling. The same process was proposed for *C. supressalis* challenged with the entomopathogenic fungus *Beauveria bassiana* [19]. In the present study, we did not perform any molecular or biochemical detection of DA in ticks’ hemocytes, only the fluorescence assay. Despite this, our imaging results suggested that receptor occupancy by the antagonist negatively impacted the DA production response (Figure 8 from Wu et al. [19]), contributing to the reduction in the phagocytic activity.

A recent study on *Ixodes ricinus* reported that the hemocytes of this tick could phagocytize *M. robertsii* conidia within two hours [9]. In the present study, *R. microplus* hemocytes were also able to phagocytose *M. anisopliae* conidia after the same time interval. Wu et al. in 2015 [19] showed a decrease in the phagocytic index (%) of *C. suppressalis* hemocytes using the antagonist SCH 23390 in the presence of bacteria *Escherichia coli*. In the same way, here, the results of the phagocytic indexes of *R. microplus* hemocytes exposed to *S. cerevisiae* or *M. anisopliae* were lower in the presence of the same antagonist (Figure 1G). Accordingly, the results obtained here reinforce the involvement of DA in the phagocytosis of external agents, as observed in insects. Furthermore, a reduction in the capacity of ticks’ hemocyte phagocytize is suggested to be linked to a lower survival capacity of the ticks after the challenge with the entomopathogenic fungus *M. anisopliae*.

## 5. Conclusions

The present study suggests the influence of DA in the phagocytosis process of hemocytes from *R. microplus*. The inoculation of a DA receptor antagonist reduces the survival, phagocytic index, and egg production index of *R. microplus* infected with *M. anisopliae*. 

## Data Availability

Not applicable.

## Supplementary Materials

The following supporting information can be downloaded at https://www.mdpi.com/article/10.3390/jof8121312/s1, Table S1: Average and standard error of initial female weight, egg mass weight, egg production index (EPI), nutritional index (NI)**,** reproductive efficiency (RE), and tick control percent of *Rhipicephalus microplus* females inoculated with the antagonist SCH 23390 (1 nM or 1 µM) and *Metarhizium anisopliae* (1 × 10^7^ conidia/mL).
