# Supplementary material for: Inhibition of Dopamine Activity and Response of Rhipicephalus microplus Challenged with Metarhizium anisopliae
Source: J Fungi (Basel). 2022 Dec 17;8(12):1312. doi: 10.3390/jof8121312 (PMC9785602; doi:10.3390/jof8121312)
Supplement: Supplementary file 1 [file jof-08-01312-s001.zip › Table S1.pdf]

Table S1. Average and standard error of initial female weight, egg mass weight, egg production index (EPI), nutritional index (NI), reproductive efficiency (RE), and tick control percent of *Rhipicephalus microplus* females inoculated with the antagonist SCH 23390 (1 nM or 1 μM) and *Metarhizium anisopliae* (1 × 10^7 conidia/mL).

	**Initial female weight (mg)**	**Egg mass weight (mg)**	**EPI (%)**	**NI (%)**	**RE (%)**	**Tick Control (%)**
**Controle**	199.4 a ± 0.01	99.2 a ± 0.008	49.7 a ± 1.5	64.4 a ± 1.9	45.7 a ± 1.9	-
**PBS**	198.0 a ± 0.01	88.2 a ± 0.008	45.1 a ± 2.8	59.4 a ± 3.4	38.1 a ± 3.4	-
**SCH A**	197.4 a ± 0.01	89.1 a ± 0.007	45.3 a ± 2.4	64.8 a ± 3.6	29.9 a ± 3.5	32.4
**SCH B**	197.5 a ± 0.01	80.6 a ± 0.008	40.7 a ± 3.1	60.7 a ± 2.7	29.5 a ± 3.1	25.2
**MA**	198.3 a ± 0.01	40.1 b ± 0.007	25.1 b ± 5.0	24.8 b ± 3.7	7.7 b ± 2.7	78.2
**SCH A + MA**	191.7 a ± 0.01	19.1 b ± 0.005	9.6 c ± 2.7	25.3 b ± 5.9	6.6 b ± 2.5	79.2
**SCH B + MA**	200.3 a ± 0.01	17.5 b ± 0.005	8.8 c ± 2.7	23.8 b ± 5.6	4.0 b ± 1.9	90.5

Means followed by the same letter, in the same column, do not differ significantly from each other ($P \geqslant 0.05$). **CTR**: untreated ticks; **PBS**: ticks inoculated with phosphate buffer solution; **SCH A**: ticks inoculated with the antagonist at 1 nM; **SCH B**: ticks inoculated with antagonist at 1 μM; **MA**: ticks inoculated with *M. anisopliae*; **SCH A + MA**: ticks inoculated with the lowest concentration of the antagonist and then the fungus; **SCH B + MA**: ticks inoculated with the highest concentration of the antagonist and then the fungus.
